# Effects of interventional lung assist on haemodynamics and gas exchange in cardiopulmonary resuscitation: a prospective experimental study on animals with acute respiratory distress syndrome

**DOI:** 10.1186/cc7716

**Published:** 2009-02-11

**Authors:** Günther Zick, Dirk Schädler, Gunnar Elke, Sven Pulletz, Berthold Bein, Jens Scholz, Inéz Frerichs, Norbert Weiler

**Affiliations:** 1 Department of Anesthesiology and Intensive Care Medicine, University Medical Center Schleswig-Holstein, Campus Kiel, Arnold-Heller-Straße 3, D-24105 Kiel, Germany

## Abstract

**Introduction:**

Interventional lung assist (ILA), based on the use of a pumpless extracorporeal membrane oxygenator, facilitates carbon dioxide (CO_2_) elimination in acute respiratory distress syndrome (ARDS). It is unclear whether an ILA system should be clamped during cardiopulmonary resuscitation (CPR) in patients with ARDS or not. The aim of our study was to test the effects of an ILA on haemodynamics and gas exchange during CPR on animals with ARDS and to establish whether the ILA should be kept open or clamped under these circumstances.

**Methods:**

The study was designed to be prospective and experimental. The experiments were performed on 12 anaesthetised and mechanically ventilated pigs (weighing 41 to 58 kg). One femoral artery and one femoral vein were cannulated and connected to an ILA. ARDS was induced by repeated bronchoalveolar lavage. An indwelling pacemaker was used to initiate ventricular fibrillation and chest compressions were immediately started and continued for 30 minutes. In six animals, the ILA was kept open and in the other six it was clamped.

**Results:**

Systolic and mean arterial pressures did not differ significantly between the groups. With the ILA open mean ± standard deviation systolic blood pressures were 89 ± 26 mmHg at 5 minutes, 71 ± 28 mmHg at 10 minutes, 63 ± 33 mmHg at 20 minutes and 83 ± 23 mmHg at 30 minutes. The clamped ILA system resulted in systolic pressures of 77 ± 30 mmHg, 90 ± 23 mmHg, 72 ± 11 mmHg and 72 ± 22 mmHg, respectively. In the group with the ILA system open, arterial partial pressure of CO_2 _was significantly lower after 10, 20 and 30 minutes of CPR and arterial partial pressure of oxygen was higher 20 minutes after the onset of CPR (191 ± 140 mmHg versus 57 ± 14 mmHg). End-tidal partial pressure of CO_2 _decreased from 46 ± 23 Torr (ILA open) and 37 ± 9 Torr (ILA clamped) before intervention to 8 ± 5 Torr and 8 ± 10 Torr, respectively, in both groups after 30 minutes of CPR.

**Conclusions:**

Our results indicate that in an animal model of ARDS, blood pressures were not impaired by keeping the ILA system open during CPR compared with the immediate clamping of the ILA with the onset of CPR. The effect of ILA on gas exchange implied a beneficial effect.

## Introduction

Interventional Lung Assist (ILA) describes a technique, which uses a pumpless arteriovenous extracorporeal membrane oxygenator to facilitate carbon dioxide (CO_2_) removal. Its ability to remove CO_2 _has been well demonstrated [1-6]. The aim of the extracorporeal CO_2 _elimination by the ILA system is to decrease the minute ventilation and the peak inspiratory pressure and thereby reduce the risk of barotrauma associated with mechanical ventilation in patients with acute respiratory distress syndrome (ARDS).

The effect of ILA on oxygenation remains unclear [7-11]. In contrast to a veno-venous extracorporeal membrane oxygenation the effect on oxygenation is limited because in the setting of an arteriovenous shunt, oxygen (O_2_) provided by the ILA system is added to the arterial blood where the saturation is already relatively high. In a previous study in a non-arrest model, we found a significant but only small effect of ILA on arterial partial pressure of O_2 _(PaO_2_) [12].

An effective operation of the ILA system relies on an arteriovenous shunt and for that reason a patient is required to have stable circulation because the blood pressure of the patient is the driving force of the device. If cardiopulmonary resuscitation (CPR) is performed in a patient treated with ILA for ARDS not only does the cardiac arrest have to be dealt with but also the severely impaired gas exchange and usually high levels of positive end-expiratory pressure (PEEP). In such a situation we found it difficult to decide whether to leave the ILA system open to take advantage of the beneficial effects described above or to clamp it and avoid the shunt with its potentially harmful effects on circulation. This has not yet been examined, so we set up an experimental model as close to the clinical situation as possible to study this effect.

Our hypothesis was that in CPR the ILA system had no significant effect on gas exchange (PaO_2 _and arterial partial pressure of CO_2_(PaCO_2_)) and a harmful effect on circulation (coronary perfusion pressure (CPP), systolic arterial pressure and mean arterial pressure).

The primary study end points were the CPP for haemodynamic stability and PaO_2 _and PaCO_2 _for gas exchange. Secondary study end points were systolic and mean arterial pressures, end-tidal partial pressure of CO_2_(PCO_2_), flow through the ILA system and return of spontaneous circulation.

## Materials and methods

The study was approved by the Committee for Animal Care of the Christian Albrechts University, Kiel, Germany, and adhered to the guidelines on animal experimentation. The experiments were performed on 12 domestic pigs (Deutsches Landschwein; Institute of Animal Breeding and Husbandry, Christian Albrechts University, Kiel, Germany) with a body weight of 41 to 58 kg. After premedication with azaperon (8 mg/kg (stresnil^®^; Janssen Cilag, Neuss, Germany)) and atropin (0.1 mg/kg (atropinsulfat^®^; B. Braun, Melsungen, Germany)) anaesthesia was induced with ketamine (5 mg/kg (ketanest^® ^S; Pfizer, Berlin, Germany)), sufentanil (0.2 μg/kg (sufenta^®^; Janssen Cilag, Germany)) and propofol (1 mg/kg (propofol-^®^Lipuro 2%; B. Braun, Melsungen, Germany)). Intubation and controlled ventilation with an inspired fraction of oxygen (FiO_2_) of 100% were performed (Siemens servo 900c ventilator, Siemens-Elema, Solna, Sweden). Anaesthesia was continued with propofol (6 to 8 mg/kg per hour) and sufentanil (10 μg/kg per hour). Lactated Ringer's solution was infused at a rate of 20 ml/kg per hour.

The carotid artery was cannulated and this line was used to draw arterial blood samples. The samples were processed by a blood gas analyser (ABL System 615, Radiometer Medical Inc., Copenhagen, Denmark). The internal jugular vein was cannulated and a catheter inserted for measurement of the central venous pressure (CVP). The contralateral internal jugular vein provided access for the placement of a pacemaker electrode. A 7 Fr pulmonary artery catheter (Arrow International, Everett, MA, USA) was inserted through the iliac artery into the thoracic descending aorta for measurement of blood pressure. PCO_2 _in respired gas, airway pressures, arterial venous pressure and CVP were monitored using the S/5 anaesthesia monitoring system (Datex Ohmeda, Helsinki, Finland).

The iliac artery and vein were cannulated with ultrasound guidance and a 13 Fr cannula was inserted into the artery and a 15 Fr cannula into the vein using Seldinger's technique. The ILA device (Novalung, Hechingen, Germany) was filled with saline solution and connected with these two cannulae, thereby generating the arteriovenous shunt required for the intended gas exchange. Five thousand units of heparin were given after the instrumentation was completely set up and the extracorporeal flow was started without oxygen flow at that time.

Acute lung injury was then induced with repeated bronchoalveolar lavages with warm saline solution, 1.5 L each. They were performed until PaO_2 _remained stable below 100 Torr with an FiO_2 _of 100% and PEEP of 5 cmH_2_O for 30 minutes.

Having achieved stable lung injury, oxygen flow through the ILA device was commenced with 10 L/minute. A low flow pressure volume (PV) manoeuvre using a slow inflation up to 30 cmH_2_O was then performed. It showed lower inflection points of more than 20 cmH_2_O indicating that PEEP values at or slightly above that level were required. Because no data exist on the best PEEP level in patients with ARDS during CPR, we chose to avoid PEEP in that high range and set PEEP arbitrarily to 12 cmH_2_O as a compromise. Ventilation was performed in the volume-controlled mode with a tidal volume of 10 ml/kg and the rate set to achieve normal arterial CO_2 _tension.

A fibrillator (Fibrillator Fi 10 M, Stöckert Instrumente, München, Germany) was then connected with the indwelling pacemaker and ventricular fibrillation was induced with the application of 10 V. Manual chest compressions were started without delay and continued for 30 minutes. In six animals, the ILA system was clamped immediately; in the other group of six animals it remained open. Adrenaline was administered as a continuous infusion at a rate of 1 μg/kg/minute with additional boluses of 1 or 3 mg if the mean blood pressure fell below 50 mmHg to ensure sufficient blood pressure and, therefore, CPP. Blood samples were drawn before fibrillation and at 5, 10, 20 and 30 minutes after onset of resuscitation. Arterial blood pressures and CVP were continuously recorded with a sampling rate of 300 Hz (ICUpilot, version 2.0, CMA/Microdialysis, Solna, Sweden). End-tidal PCO_2 _and flow through the ILA system were registered at 5, 10, 20 and 30 minutes. The chest compressions were stopped after 30 minutes and defibrillation was performed with 300 Joule (Lifepak 12, Physio-control, Medtronic, Redmond, WA, USA). Restoration of spontaneous circulation was intended. In cases where it was not successful after three attempts, no further resuscitation was performed.

### Statistical analysis

The results are presented as mean values ± standard deviations. Statistical analysis was performed using GraphPad Prism version 4.03 for Windows (GraphPad Software, San Diego, CA, USA). Two-way analysis of variance followed by the Bonferroni multiple comparison test was applied to test the significance of differences between the measurements. Statistical significance was accepted at p < 0.05. The reported P values are two-tailed.

## Results

Before initiation of resuscitation all animals had a severe lung injury and a stable haemodynamic situation with a systolic arterial blood pressure of 113 ± 13 mmHg in the group in which ILA would be kept open and 117 ± 11 mmHg in the group that would have ILA clamped. The corresponding mean arterial pressures were 89 ± 7 mmHg and 77 ± 8 mmHg, respectively. These blood pressures generated a flow through ILA of 1.7 ± 0.3 L/minute. After lung injury, PaO_2 _in the open group stabilised at a level of 123 ± 25 Torr and 124 ± 37 Torr in the other group.

**Figure 1:**
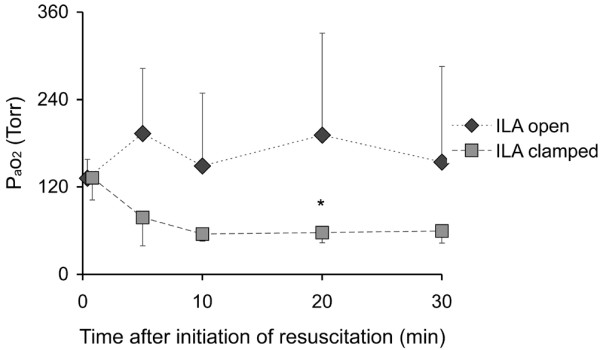
Arterial partial pressure of oxygen (PaO_2_) in the course of resuscitation. ILA = interventional lung assist. * p < 0.05.

Performing the PV manoeuvre after the induction of ARDS and before CPR showed lower inflection points of 19 ± 5 cmH_2_O. Setting the PEEP 2 cmH_2_O above the respective lower inflection point resulted in an increase of PaO_2 _to 430 ± 106 Torr and 407 ± 132 Torr in the two groups. After reduction of PEEP to 12 mmHg before initiating circulatory arrest and CPR, PaO_2 _fell to 132 ± 26 Torr and 133 ± 31 Torr (Figure 1).

When we tried to determine the CVP and hence the CPP during offline analysis, we found that the interpretation could not be performed reliably because of artefacts in the CVP readings caused by the chest compression during CPR.

**Figure 2:**
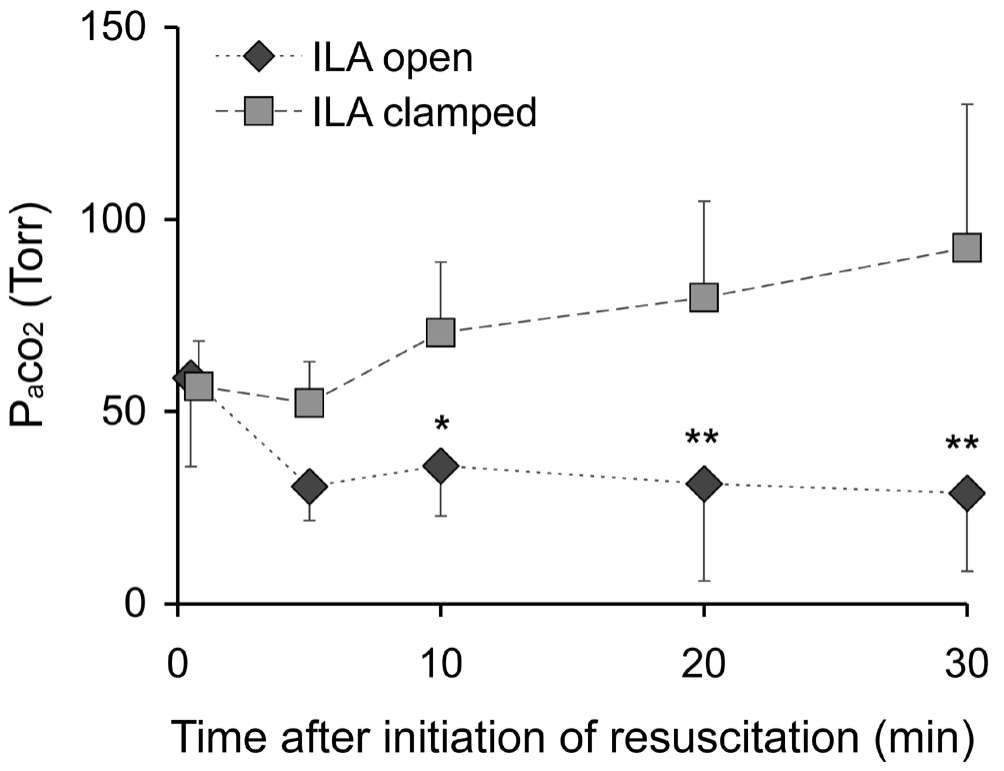
Arterial partial pressure of carbon dioxide (PaCO_2_) in the course of resuscitation. ILA = interventional lung assist. * p < 0.05; ** p < 0.005.

PaCO_2 _was significantly lower in the group with the ILA system open (Figure 2). PaO_2 _was higher in this group, however, the difference was only significant at 20 minutes (Figure 1).

**Figure 3:**
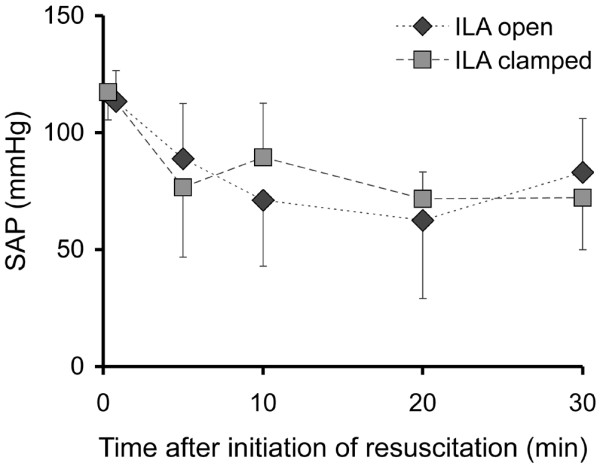
Systolic arterial pressure (SAP) in the course of resuscitation. ILA = interventional lung assist.

**Figure 4:**
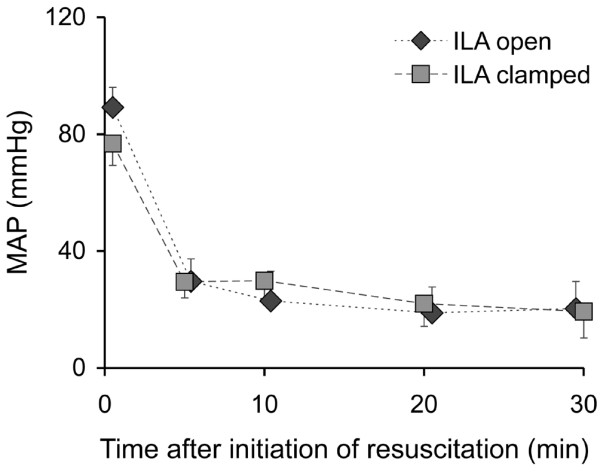
Mean arterial pressure (MAP) in the course of resuscitation. ILA = interventional lung assist.

With chest compressions and with ILA open, systolic blood pressures of 89 ± 26 mmHg at 5 minutes, 71 ± 28 mmHg at 10 minutes, 63 ± 33 mmHg at 20 minutes and 83 ± 23 mmHg at 30 minutes could be achieved (Figure 3). With ILA clamped, the following pressures were determined: 77 ± 30 mmHg, 90 ± 23 mmHg, 72 ± 11 mmHg and 72 ± 22 mmHg, respectively. Mean blood pressures were 30 ± 7 mmHg in the group with ILA open and 30 ± 6 mmHg in the group with ILA clamped at five minutes, decreasing continuously to 20 ± 9 mmHg with ILA open and 19 ± 9 mmHg with ILA clamped at 30 minutes (Figure 4).

**Figure 5:**
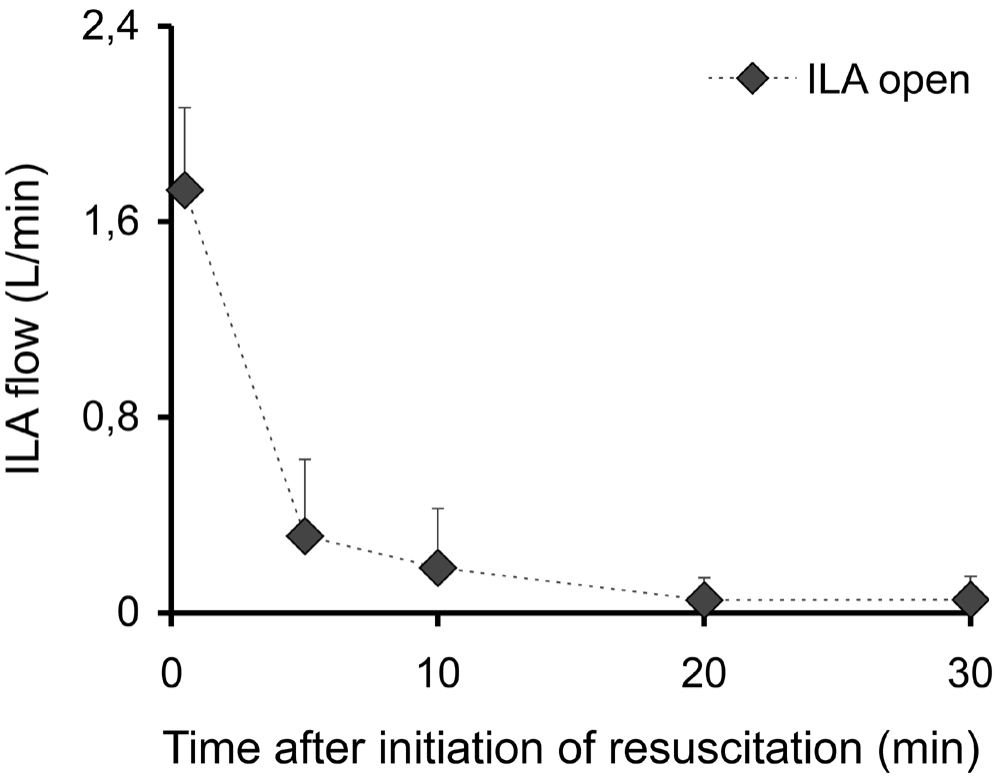
Flow through the interventional lung assist (ILA) device in the course of resuscitation.

An adrenaline dose of 3.3 ± 2.7 mg in the group with ILA open and 3.2 ± 0.8 mg in the group with ILA clamped was given at five minutes, at 10 minutes the cumulative dose was 6.5 ± 3.3 mg and 7.5 ± 1.8 mg, and at 20 minutes 13.7 ± 7.0 mg and 13.2 ± 4.3 mg, respectively, was given. The total dose of adrenaline after 30 minutes was about 19 mg in each group (18.8 ± 8.6 mg with ILA open and 18.7 ± 6.2 mg with ILA clamped). Flow through the ILA system decreased under conditions of resuscitation (Figure 5). In three cases a flow reversal was observed at the end of the observation time, seen as a change in the blood colour at the inlet and outlet of the ILA. At the same time, negative flow values in a range below 0.02 L/minute were detected.

Neither blood pressures nor the administered dose of adrenaline were significantly different between the groups.

**Figure 6:**
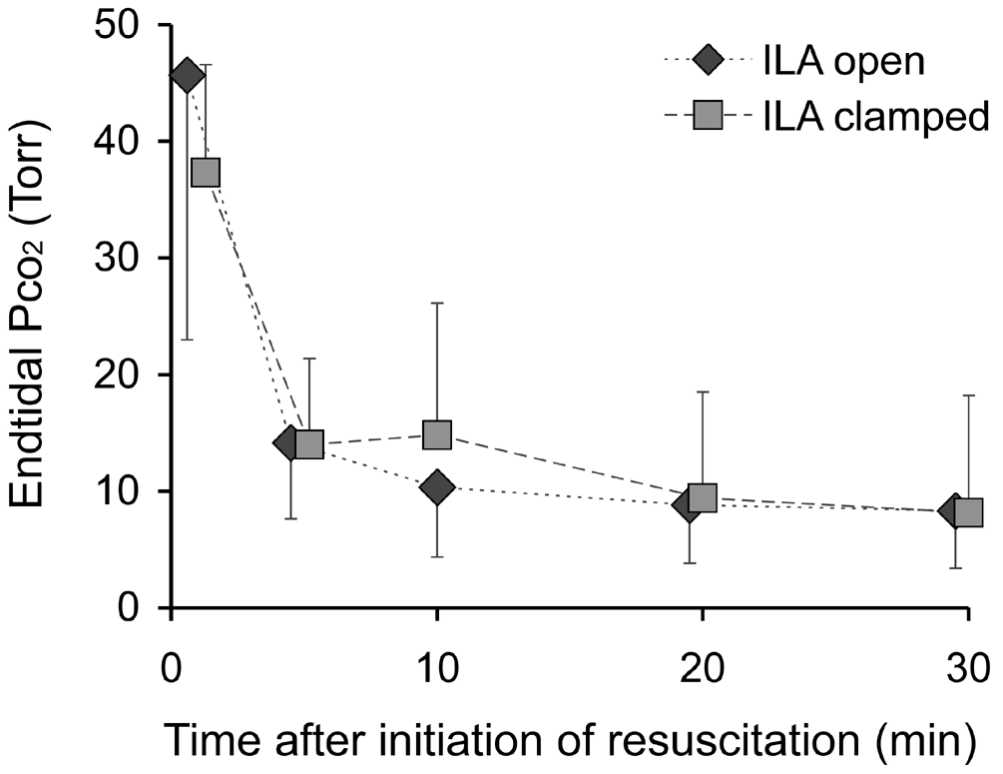
End-tidal partial pressure of carbon dioxide (CO_2_) in the course of resuscitation. ILA = interventional lung assist.

End-tidal PCO_2 _decreased from 46 ± 23 Torr with ILA open and 37 ± 9 Torr with ILA clamped before resuscitation to 8 ± 5 Torr and 8 ± 10 Torr, respectively, at the end of 30 minutes of CPR and was not different between the groups (Figure 6).

Return of spontaneous circulation did not occur in either group after 30 minutes of CPR.

## Discussion

The use of extracorporeal lung assist is an additional therapeutic approach in patients with severe ARDS that facilitates a lung protective ventilation strategy. This is achieved mainly by an extracorporeal CO_2 _elimination and possibly sustained by a small oxygenation effect generated by an arteriovenous shunt through an artificial membrane.

In the case of CPR in a patient with severe ARDS and established extracorporeal lung assist, the question arises whether ILA should be kept open or clamped. In such a situation the extracorporeal lung assist may still exert its beneficial effects on gas exchange or it may be harmful because of the arteriovenous shunt it causes. We have tested the effects of CPR on circulation and gas exchange with or without an ILA device operating in animals with ARDS.

Before initiation of resuscitation all animals had a severe ARDS and a stable haemodynamic situation. After induction of ventricular fibrillation chest compressions were started without delay. Our primary goal was not the survival after prolonged ischaemia, so we did not adhere to the Utstein Guidelines with the recommended 'non-intervention interval' [13]. Our model was designed to resemble an ARDS patient in an ICU. CPR would be started without delay in that setting.

We could not analyse the CVP reliably, which prevented the intended analysis of the CPP. This was due to the fact that we intended to analyse the CPP offline and only then recognised the invalid CVP measurement after the experiments were completed. Therefore, we took the more robust arterial pressure readings to assess the effects of ILA on circulation. The blood pressure that could be generated with chest compressions did not differ significantly between the two groups (Figures 3 and 4). End-tidal CO_2 _was also in the same range (Figure 6). Therefore, we assume that the circulation did not differ significantly and that the shunt by the ILA did not deteriorate the circulation.

Because of the low arterial pressure, flow through the ILA system decreased and fell to almost zero in the course of the 30-minute resuscitation period (Figure 5). This is consistent with the differences in PaCO_2 _(Figure 2) and PaO_2 _(Figure 1) also occurring in the early phase of CPR and a continuously decreasing contribution of the ILA in the further course of CPR.

Adrenaline was administered according to the arterial blood pressure and our goal was to keep the mean pressure above 50 mmHg according to guidelines that would be applied in a clinical situation [14] which recommend 1 mg of adrenaline every three to five minutes. We adjusted the dose when the arterial pressure did not respond according to our protocol. The response to our adrenaline therapy might have additionally been blunted by a systemic inflammatory response syndrome caused by repeated lung lavages.

Behringer and colleagues found that high doses of adrenaline were associated with unfavourable neurological outcome but restoration of spontaneous circulation was possible with increasing cumulative doses of adrenaline. In his conclusion he suggested that further investigations should be attempted to better define limits for adrenaline doses during CPR [15].

The resuscitation was continued for 30 minutes without any attempt at defibrillation. First defibrillation was performed after 30 minutes. In neither group, return of spontaneous circulation could be established. As our intention was to examine the effect of ILA on haemodynamics and gas exchange over a sufficient time interval, we may have missed the point where an effect on the survival may have been discernible. The main reasons for the lack of survival may therefore be the long duration of CPR, the severity of the induced lung injury and relatively low arterial blood pressure. All animals had severe ARDS, which may have caused a systemic inflammatory response syndrome with impaired responsiveness to adrenaline. Redberg and colleagues reported arterial blood and end-tidal CO_2 _pressures comparable with our data in 20 patients from whom five were successfully resuscitated [16]. Other authors report even lower arterial pressures and ensuing successful resuscitation; however, with much shorter resuscitation time and no accompanying ARDS [17].

Another factor negatively affecting the response to attempted defibrillation after 30 minutes of CPR was probably the relatively high intrathoracic pressure. The interpretation of our low flow PV recruitment manoeuvre would have indicated that high PEEP levels of over 20 cmH_2_O would have been required. We are not aware of any recommendation for PEEP setting in patients or animals with ARDS during CPR. Therefore, we chose to set PEEP at 12 cmH_2_O as a compromise between derecruitment of aerated lung regions and impairment of circulation. Many authors were able to demonstrate the harmful effect of high intrathoracic pressures in CPR [18-22]. As a consequence, Aufderheide and colleagues found increased survival rates with reduced intrathoracic pressures in CPR after cardiac arrest using an impedance threshold device [23].

The main limitations of our study are the missing data on the CPP and other measures of tissue perfusion. Another limitation of our study is the deliberate decision to set the PEEP level at 12 cmH_2_O. However, no data are available at present on how the optimal PEEP should be set in this situation.

## Conclusions

The blood pressures were not impaired by keeping the ILA system open during CPR compared with the immediate clamping of the ILA with the onset of CPR and PaO_2 _and PaCO_2 _showed a potential benefit from the open ILA system. We therefore conclude that when in doubt the ILA system should be kept open. We found no evidence suggesting that ILA should be clamped. The optimal PEEP setting in CPR in ARDS patients remains unclear and requires further studies.

## Key messages

• Our experimental study indicates that ILA does not interfere with haemodynamics in CPR.

• ILA may have beneficial effects on gas exchange during CPR.

## Abbreviations

ARDS: acute respiratory distress syndrome; CO_2_: carbon dioxide; CPR: cardiopulmonary resuscitation; CPP: coronary perfusion pressure; CVP: central venous pressure; FiO_2_: inspired fraction of oxygen; ILA: interventional lung assist; O_2_: oxygen; PaCO_2_: arterial partial pressure of carbon dioxide; PaO_2_: arterial partial pressure of oxygen; PCO_2_: partial pressure of carbon dioxide; PEEP: positive end-expiratory pressure; PV: pressure volume.

## Competing interests

The study was partially supported by Novalung, Hechingen, Germany.

## Authors' contributions

GZ participated in design of the study, carried out the study and drafted the manuscript. DS carried out the study and participated in the analysis of data. GE carried out the study and participated in the analysis of data. SP carried out the study. BB participated in the design of the study and revised the manuscript. JS participated in design and coordination. IF performed the analysis and interpretation of the data and revised the manuscript. NW conceived the study and participated in the design of the study, analysis and interpretation of data and revision of the manuscript. All authors read and approved the final manuscript.
